# Supplementary material for: The New Dipeptide TSPO Ligands: Design, Synthesis and Structure–Anxiolytic Activity Relationship
Source: Molecules. 2020 Nov 4;25(21):5132. doi: 10.3390/molecules25215132 (PMC7663781; doi:10.3390/molecules25215132)
Supplement: Supplementary file 1 [file molecules-25-05132-s001.pdf]

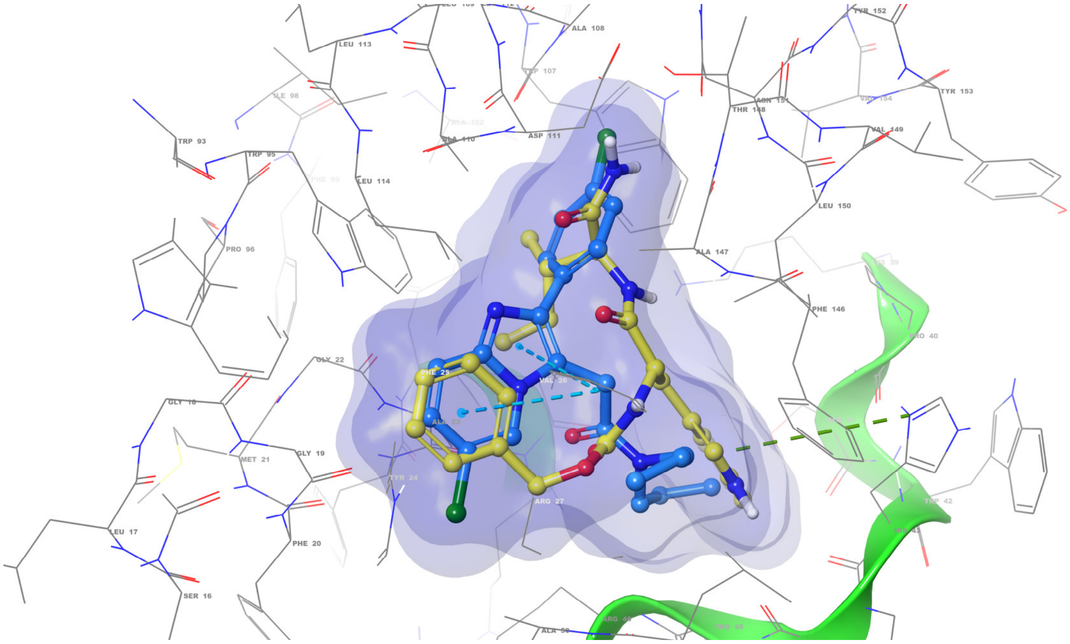

Figure S1. The superposition of alpidem and GD-23 conformations docked in TSPO. Yellow – GD-23. blue - alpidem. TSPO contact residues are shown with wire representation (visualization using Maestro).

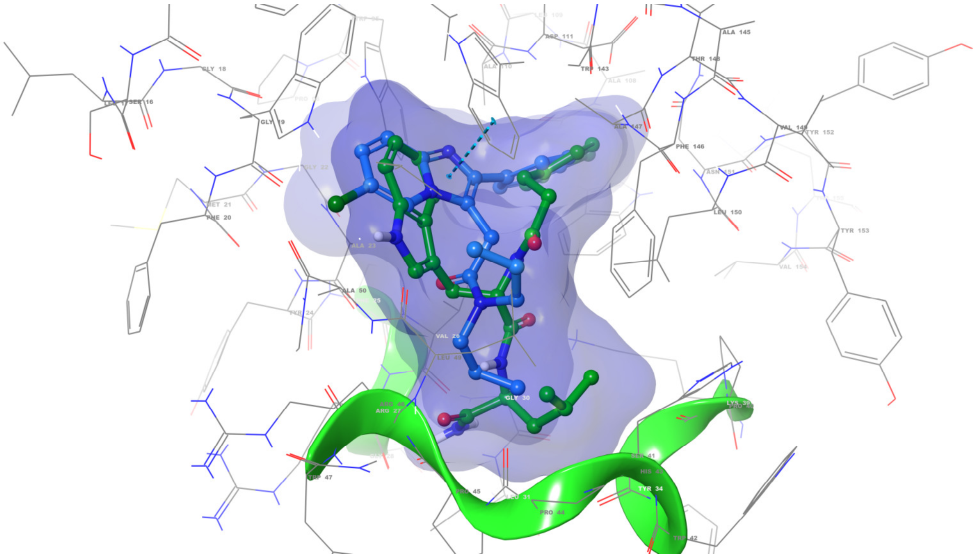

Figure S2. The superposition of alpidem and GD-102 conformations docked in TSPO. Green - GD-102. blue - alpidem. TSPO contact residues are shown with wire representation (visualization using Maestro).

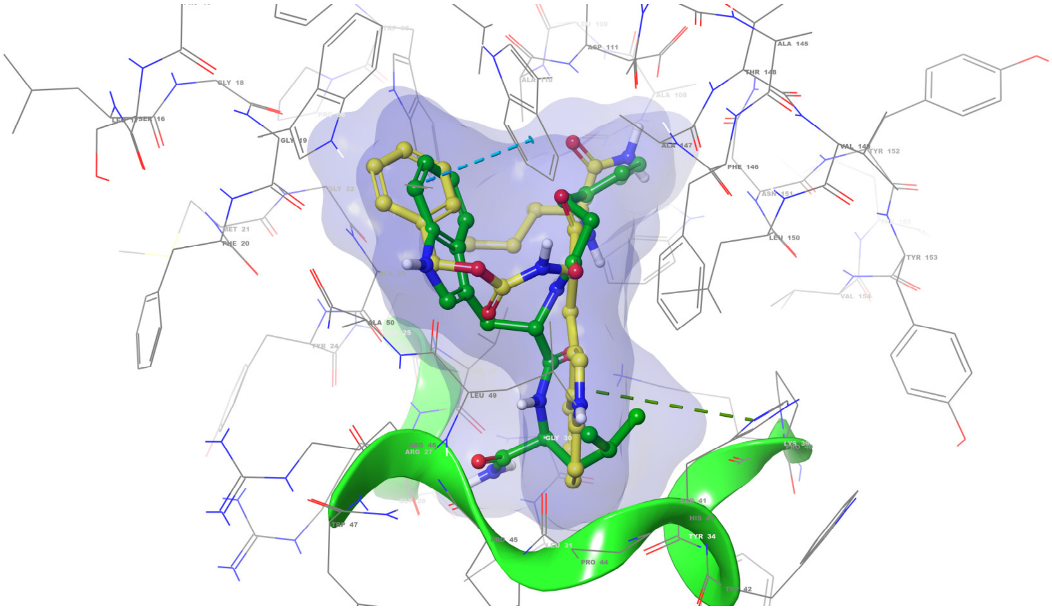

Figure S3. The superposition of GD-23 and GD-102 conformations docked in TSPO. Yellow - GD-23. green - GD-102. TSPO contact residues are shown with wire representation (visualization using Maestro).

Table S1. Glide Score and Glide emodel data for all synthesized dipeptides in compare with alpidem and known TSPO inhibitor PK11195

Ligand	Glide gscore	Glide emodel. kcal/mol
Alpidem	-8.89	-66.73
PK-11195	-8.08	-59.16
GD-102	-9.60	-71.97
GD-107	-8.75	-57.08
GD-108	-8.77	-44.05
GD-123	-9.03	-65.85
GD-125	-8.69	-69.04
GD-128	-9.15	-51.67
GD-129	-8.06	-66.39
GD-23	-8.76	-52.85

Table S2. Effect of dipeptide GD-102 (i.p.) on the ICR mice behavior in the Elevated Plus-Maze test.

Dose, mg/kg, i.p.	Time spent in open arms, sec	Time spent in closed arms, sec	Number of entries in open arms	Number of entries in closed arms	Time spent in open arms, %	Time spent in closed arms, %	Total number of entries in the arms	Relative time spent in open arms, %	Relative number of entries in open arms, %
Control (water)	2,5 (0,0;11,0)	264,5 (246,5;272,0)	0,5 (0,0;2,0)	9,0 (7,5;10,5)	0,8 (0,0;3,7)	88,2 (82,2;90,7)	9,0 (7,5;11,5)	0,9 (0,0;4,3)	4,2 (0,0;15,3)
0,1	42,5* (10,0;95,0)	224,0* (170,5;246,0)	2,0 (1,5;3,0)	10,5 (7,5;12,5)	14,2* (3,3;31,7)	74,7* (56,8;82,0)	13,5* (11,0;14,0)	16,7* (3,9;34,1)	16,2 (10,7;28,2)
0,5	65,5* (23,0;85,0)	195,5* (181,0;236,5)	3,0* (1,0;4,5)	9,0 (8,0;9,5)	21,8* (7,7;28,3)	65,2* (60,3;78,8)	12,0* (10,5;14,5)	25,9* (8,9;30,9)	22,5* (10,6;33,9)
1,0	49,5* (25,0;60,0)	209,5* (194,0;239,0)	3,0 (1,0;5,0)	7,0 (6,0;8,5)	16,5* (8,3;20,0)	69,8* (64,7;79,7)	11,0 (8,0;13,0)	19,1* (9,2;23,9)	27,4 (11,7;43,6)
p (Kruskal-Wallis test)	0.0106	0.0080	0.0947	0.1128	0.0106	0.0080	0.0728	0.0102	0.1050

Notes: Data are represented as Me (q25;q75); number of animals = 8 in each group.

* – $p < 0.05$, statistically significant differences compared with the control group under the Mann-Whitney test.

Table S3. PK11195 cancelled the anxiolytic effect of GD-102 in the Elevated Plus-Maze test in ICR mice.

Experimental groups	Time spent in open arms, sec	Time spent in closed arms, sec	Number of entries in open arms	Number of entries in closed arms	Time spent in open arms, %	Time spent in closed arms, %	Total number of entries in the arms	Relative time spent in open arms, %	Relative number of entries in open arms, %
Control water i.p. + water i.p.	29,0 (23,5;34,0)	228,5 (218,5;240,0)	2,0 (1,5;2,5)	8,0 (7,0;9,5)	9,7 (7,8;11,3)	76,2 (72,8;80,0)	9,5 (9,0;11,5)	11,2 (9,1;12,9)	17,7 (13,2;26,7)
PK11195 10 mg/kg i.p. + water i.p.	24,0 (12,0;42,0)	226,0 (207,5;245,0)	2,0 (1,0;3,0)	10,0 (7,5;12,5)	8,0 (4,0;14,0)	75,3 (69,2;81,7)	12,5 (9,5;15,5)	10,1 (4,6;16,1)	20,0 (10,4;23,6)
GD-102 0,5 mg/kg i.p. + water i.p.	116,5* (87,0;161,5)	136,0* (103,0;157,0)	5,5* (3,5;7,5)	6,5 (5,0;8,5)	38,8* (29,0;53,8)	45,3* (34,3;52,3)	11,5 (11,0;14,5)	46,1* (35,5;61,4)	44,9* (39,6;54,3)
PK11195 10,0 mg/kg i.p. + GD-102 0,5 mg/kg i.p.	24,5 (17,5;36,0)	221,5 (219,0;254,5)	2,5 (1,5;3,5)	10,0 (9,0;11,0)	8,2 (5,8;12,0)	73,8 (73,0;84,8)	13,0 (11,0;14,0)	10,2 (6,6;13,5)	22,3 (13,6;29,7)
p (Kruskal-Wallis test)	0.0140	0.0139	0.0308	0.1003	0.0140	0.0139	0.2600	0.0123	0.0155

Notes: Data are represented as Me (q25;q75); number of animals = 8 in each group.

* – $p < 0.05$, statistically significant differences compared with the control group under the Mann-Whitney test.

Two-way ANOVA results with Tukey's multiple comparisons test for the data from table S3.

Table S4.Time spent in open arms, s

Pairwise comparisons	p values
GD-102 vs H_2O	0,0032
PK11195 vs H_2O	0,9953
GD-102+PK11195 vs H_2O	0,9970
GD-102 vs PK11195	0,0017

GD-102 vs PK11195+GD-102	0,0053
PK11195 vs PK11195+GD-102	0,9711

Table S5. Time spent in center, s

Pairwise comparisons	p values
GD-102 vs H_2O	0,9849
PK11195 vs H_2O	0,9977
GD-102+PK11195 vs H_2O	0,9358
GD-102 vs PK11195	0,9983
GD-102 vs PK11195+GD-102	0,7860
PK11195 vs PK11195+GD-102	0,8679

Table S6. Time spent in closed arms, s

Pairwise comparisons	p values
GD-102 vs H_2O	<0,0001
PK11195 vs H_2O	0,9999
GD-102+PK11195 vs H_2O	0,9641
GD-102 vs PK11195	<0,0001
GD-102 vs PK11195+GD-102	<0,0001
PK11195 vs PK11195+GD-102	0,9469

Table S7. Number of entries in open arms, n

Pairwise comparisons	p values
GD-102 vs H_2O	0,0052
PK11195 vs H_2O	0,9935
GD-102+PK11195 vs H_2O	0,9122
GD-102 vs PK11195	0,0100
GD-102 vs PK11195+GD-102	0,0258
PK11195 vs PK11195+GD-102	0,9788

Table S8. Number of entries in closed arms, n

Pairwise comparisons	p values
GD-102 vs H_2O	0,7143
PK11195 vs H_2O	0,6565
GD-102+PK11195 vs H_2O	0,6565
GD-102 vs PK11195	0,1418
GD-102 vs PK11195+GD-102	0,1418
PK11195 vs PK11195+GD-102	>0,9999

Table S9. Time spent in open arms, %

Pairwise comparisons	p values
GD-102 vs H_2O	<0,0001
PK11195 vs H_2O	>0,9999
GD-102+PK11195 vs H_2O	0,9966
GD-102 vs PK11195	<0,0001
GD-102 vs PK11195+GD-102	<0,0001
PK11195 vs PK11195+GD-102	0,9974

Table S10. Time spent in center, %

Pairwise comparisons	p values
GD-102 vs H_2O	0,9843
PK11195 vs H_2O	0,9975
GD-102+PK11195 vs H_2O	0,9386
GD-102 vs PK11195	0,9984
GD-102 vs PK11195+GD-102	0,7886
PK11195 vs PK11195+GD-102	0,8691

Table S11. Time spent in closed arms, %

Pairwise comparisons	p values
GD-102 vs H_2O	<0,0001
PK11195 vs H_2O	0,9999
GD-102+PK11195 vs H_2O	0,9642
GD-102 vs PK11195	<0,0001
GD-102 vs PK11195+GD-102	<0,0001
PK11195 vs PK11195+GD-102	0,9465

Table S12. Total numbers of entries in the arms, n

Pairwise comparisons	p values
GD-102 vs H_2O	0,5085
PK11195 vs H_2O	0,6595
GD-102+PK11195 vs H_2O	0,5085
GD-102 vs PK11195	0,9946
GD-102 vs PK11195+GD-102	>0,9999
PK11195 vs PK11195+GD-102	0,9946

Table S13. Relative time spent in open arms , $t_{open}/t_{open}+t_{closed}$, %

Pairwise comparisons	p value

GD-102 vs H_2O	<0,0001
PK11195 vs H_2O	>0,9999
GD-102+PK11195 vs H_2O	0,9950
GD-102 vs PK11195	<0,0001
GD-102 vs PK11195+GD-102	<0,0001
PK11195 vs PK11195+GD-102	0,9978

Table S14. Relative number of entries in open arms , $n_{open}/n_{open}+n_{closed}$, %

Pairwise comparisons	p values
GD-102 vs H_2O	0,0032
PK11195 vs H_2O	0,9953
GD-102+PK11195 vs H_2O	0,9970
GD-102 vs PK11195	0,0017
GD-102 vs PK11195+GD-102	0,0053
PK11195 vs PK11195+GD-102	0,9711
